# A Review on the Application and Limitations of Administrative Health Care Data for the Study of Acute Kidney Injury Epidemiology and Outcomes in Children

**DOI:** 10.3389/fped.2021.742888

**Published:** 2021-10-27

**Authors:** Emma H. Ulrich, Gina So, Michael Zappitelli, Rahul Chanchlani

**Affiliations:** ^1^Division of Pediatric Nephrology, Department of Pediatrics, University of Alberta, Edmonton, AB, Canada; ^2^Department of Health Sciences, McMaster University, Hamilton, ON, Canada; ^3^Division of Nephrology, Department of Pediatrics, Hospital for Sick Children, University of Toronto, Toronto, ON, Canada; ^4^Institute of Clinical and Evaluative Sciences, Ontario, ON, Canada; ^5^Department of Health Research Methods, Evidence and Impact, McMaster University, Hamilton, ON, Canada; ^6^Division of Pediatric Nephrology, Department of Pediatrics, McMaster University, Hamilton, ON, Canada

**Keywords:** administrative health care data, acute kidney injury, nephrology, epidemiology, pediatrics

## Abstract

Administrative health care databases contain valuable patient information generated by health care encounters. These “big data” repositories have been increasingly used in epidemiological health research internationally in recent years as they are easily accessible and cost-efficient and cover large populations for long periods. Despite these beneficial characteristics, it is also important to consider the limitations that administrative health research presents, such as issues related to data incompleteness and the limited sensitivity of the variables. These barriers potentially lead to unwanted biases and pose threats to the validity of the research being conducted. In this review, we discuss the effectiveness of health administrative data in understanding the epidemiology of and outcomes after acute kidney injury (AKI) among adults and children. In addition, we describe various validation studies of AKI diagnostic or procedural codes among adults and children. These studies reveal challenges of AKI research using administrative data and the lack of this type of research in children and other subpopulations. Additional pediatric-specific validation studies of administrative health data are needed to promote higher volume and increased validity of this type of research in pediatric AKI, to elucidate the large-scale epidemiology and patient and health systems impacts of AKI in children, and to devise and monitor programs to improve clinical outcomes and process of care.

## Introduction

Acute kidney injury (AKI), characterized by an abrupt deterioration of kidney function, is common in adults and children, and the incidence is increasing (1). The Kidney Disease Improving Global Outcomes (KDIGO) definition incorporates rise in serum creatinine and decreased urine output to identify AKI and classify severity. Approximately 5% of hospitalized children develop AKI (2), and 20–50% of children in the intensive care unit (ICU) and those with cardiac surgery develop AKI (3, 4). Pediatric and adult AKIs are associated with adverse in-hospital outcomes, including mortality and prolonged hospitalization. Many studies among adults have shown that AKI is a strong risk factor for chronic kidney disease (CKD), end-stage kidney disease (ESKD), hypertension (HTN), cardiovascular disease, and mortality (5). Among children, available data suggest that AKI is also associated with worse long-term outcomes (6–9).

Administrative health care databases have enabled expansive observational studies because these data are pre-collected and easily accessible and provide a wealth of information regarding epidemiology, risk factors, and outcomes of AKI (10). The purpose of this review is to provide an overview of administrative health care data, as well as its strengths and limitations. In addition, we review validation studies to identify individuals with AKI using administrative health data. Finally, we provide an update on the incidence, risk factors, and outcomes after AKI, as learned from the studies conducted in the last 5 years using administrative health data.

## Administrative Health Care Data Research

**Figure 1:**
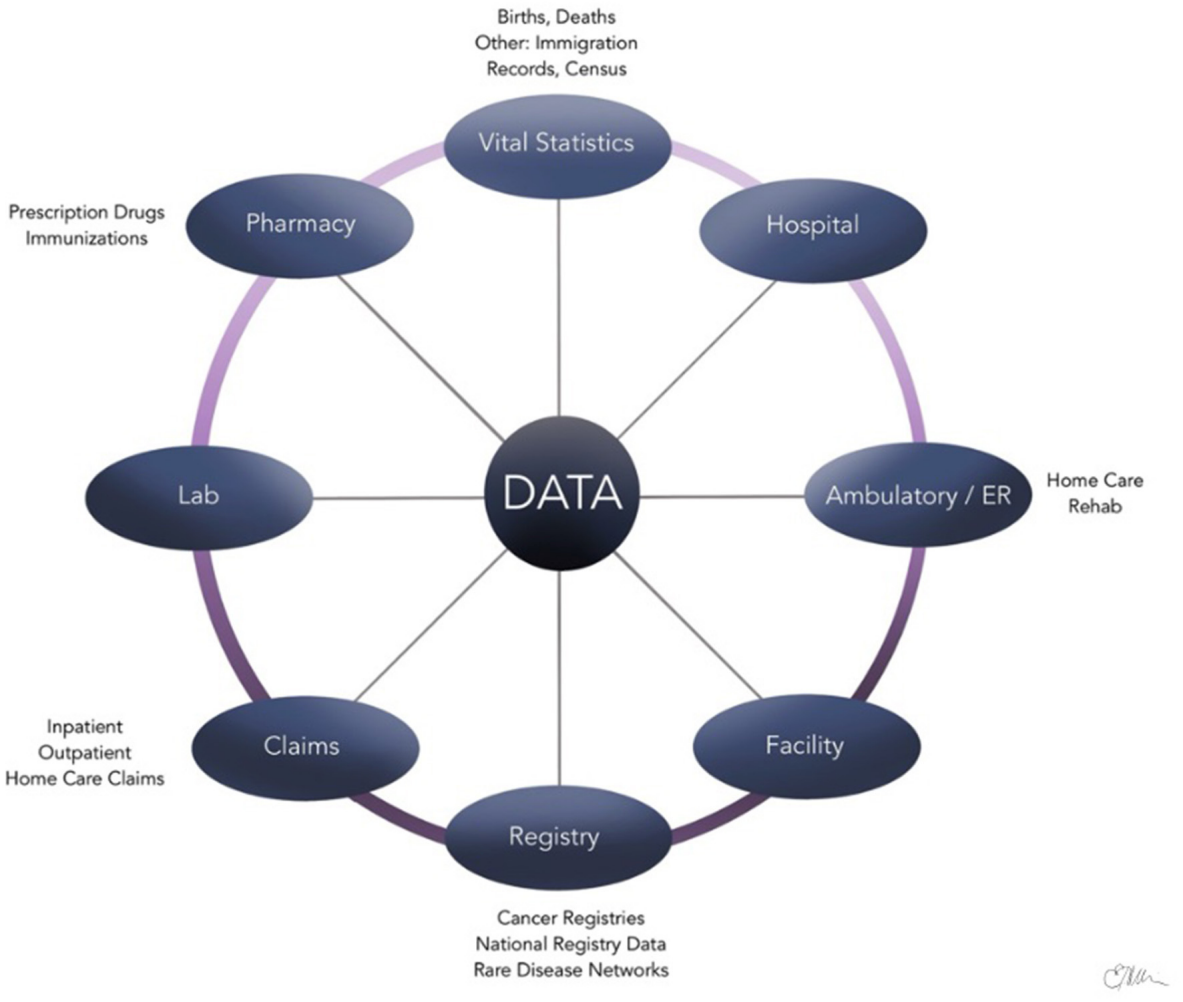
Sources of administrative health data.

Administrative health care databases store large quantities of information that are routinely compiled and updated by provinces and/or countries on patients, health care providers, and institutions during various patient encounters (11, 12). Examples of data collected are summarized in Figure 1 and include physician billing codes, prescription claims, vital records, and hospitalization/discharge summaries (13). These data contained within the administrative health databases are often referred to as “big data,” which are distinguished by the large volume of information, speed at which it is generated, and the wide range of fields that it covers (14). Epidemiological studies, in particular, benefit greatly from the availability of administrative health care databases to evaluate and track the health of large populations over a period (12). For example, the *International Classification of Diseases* (*ICD*) diagnostic codes found in the databases have been established as an international standard for classification of diseases (15, 16). Using standardized *ICD* diagnostic codes as a main source of data allows clinical epidemiologists to study patterns of disease, patient care, and various health outcomes in an efficient way (17).

**Figure 2:**
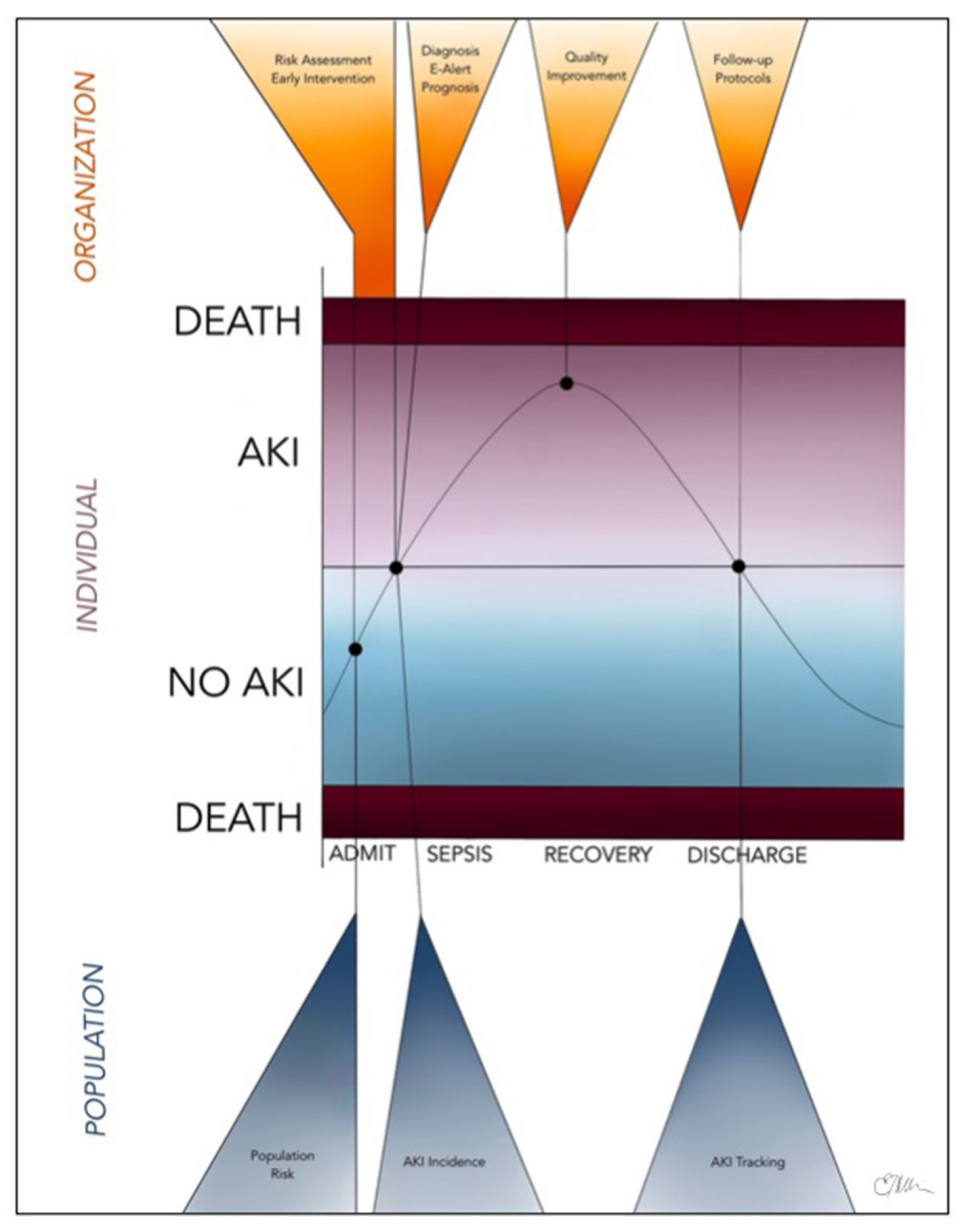
Future, real-time application of “big data” (including administrative health data and electronic medical records) to the identification, management, and monitoring of children and adults with AKI at the individual, organizational, and population levels.

In recent years, the use of administrative health data as a powerful research tool has become increasingly established and extensively applied to various fields of epidemiological research (11, 15, 16). AKI, in particular, is an ideal disease to study using administrative health data (18) due to the high prevalence in hospitalized patients, increased risk for major morbidity (CKD, progression to ESKD, and death), financial costs, and recent research demonstrating that care of patients with AKI is suboptimal due to avoidable systemic issues (18). A series of statements from the Acute Dialysis Quality Initiative (ADQI) consensus conference in 2015 outline the potential of large database research for AKI, including real-time prediction of risk for AKI, developing electronic alerts for quality improvement, and “tagging” AKI patients for longitudinal care and advancing our understanding of long-term outcomes following AKI (18–23). Currently, real-time AKI alerts using electronic systems notify health care providers using the KDIGO definition of AKI events (20). In the future, however, machine learning could incorporate real-time patient comorbidities, nephrotoxin exposure, and so on that would allow these electronic systems to provide prognostic data directly to health care providers managing the individual patient, even before an AKI event has occurred (20). The potential real-time application of “big data” to the management of AKI at the individual, organizational, and institutional level is summarized in Figure 2.

## Strengths and Limitations of Administrative Health Data Research

To effectively conduct and interpret outcomes of administrative health data research, it is important to understand the strengths and limitations (Table 1).

**Table 1:** Summary of strengths and limitations of administrative health research.

**Strengths**	**Limitations**
**Large sample size**	**Gaps in clinical information**
•Reduces sample error and biases •Provides information for addressing precise clinical research questions	•Lack of information about certain comorbidities and confounders •Changes to coding practices contribute to gaps in data
**Accessibility of data**	**Lack of validation studies**
•Addresses drawbacks of traditional RCTs •Cost-effective •Increasingly accessible by the broader research community •Ability to study rare diseases	•Gaps in literature for validation studies of administrative health data •Pediatric-focused validation studies are very limited
**Long follow-up**	**Statistical issues and biases**
•Continuous and long periods of data collection •Easy follow-up process • **Ability to link data to other data sources**	
•Other databases •Electronic medical records •Census and surveys	•Overestimated statistical significance •Information/misclassification bias •Selection bias •More expertise in analysis of administrative health databases required

### Strengths

One of the most significant advantages of administrative health care data is the very large sample size, which in turn reduces sampling error, increases statistical power, and increases validity (16, 24). The inclusion of a larger and more diverse population also increases generalizability of the study findings to a greater population (25). Having larger numbers of variables collected also allows researchers adjust for many types of variables in multivariable analyses (24).

Although prospective randomized controlled trials (RCTs) are considered to be the gold standard of scientific evidence in clinical medicine, it is often difficult to conduct them because of various logistical, financial, and ethical limitations (24). As administrative health care data are pre-collected and readily available, they allow studies to be less costly and much quicker to perform (24). In fact, cluster RCTs that obtain data through administrative health databases can act as a solution to allow for inclusion of a broad number of facilities and larger representative samples of patients (26). As with any cluster RCT, care would be required to ensure information about exposure status, and outcomes are generalizable and derived from data sources in a consistent manner across trial centers. The heterogeneity and accessibility of administrative health data also allow for easier identification of patients with rare diseases (27–29).

Administrative health care databases often allow patients to be followed over extended time periods (6, 30, 31). Unlike studies that require primary data collection, administrative health databases do not involve researchers having to contact individuals, and loss to follow-up is not a significant issue (unless the patients do not immigrate) when conducting longitudinal studies for outcomes. In fact, researchers can follow patients over a long period by linking their clinical research records to administrative databases with the patients' consent (32). This, in turn, simplifies the follow-up process and eliminates any chances of non-response and recall bias (33).

Administrative health care data also can be supplemented and linked with other data sources, allowing for a larger and more comprehensive data set. This involves the easily computerized process of linking an individual using personal identifier variables across different databases without affecting patient privacy (16). Other data sources, such as census or citizenship data, allow researchers to gain more information about certain target groups' socioeconomic status and lifestyle (34, 35). Linkage with electronic medical records, which includes more detailed information about a patient's health, helps to increase the validity of research findings (36, 37).

### Limitations

Because of health care databases being structured primarily for administrative and billing purposes, they often lack additional clinical information about the diseases of interest, health outcomes, and medications (38). Detailed data about some comorbidities, anthropometric measures, quality of life, education status, physical activity levels, and patient-reported outcomes are usually unavailable or recorded inconsistently and can negatively impact the research process (39–41). Additionally, changes in clinical diagnostic billing coding practices and different software systems that different physicians and institutions use may cause additional gaps in data (41). For example, the mandatory transition from *ICD-9* to *ICD-10* diagnostic codes in 2015 involved nearly four times more codes (42). Hence, it is important to consider the changing thresholds and other factors for diagnosing certain diseases to prevent overestimation or underestimation of cases when evaluating incidence and outcomes, which is mainly accomplished through regular and rigorous validation studies. To address this, it is essential for policymakers to devise a standard framework surrounding the coding and software system used within institutions to help bridge the data and communication gaps. This will help ensure that the collection of data is more consistent within and between countries and that the information can be used more effectively in research studies.

Patient identification in administrative health data research is usually made upon the basis of specific diagnostic or procedural codes of disease (11). Validation studies that evaluate the different combination of codes and criteria, also known as “algorithms,” are essential when conducting administrative health research, but are currently very limited (11, 43). A scoping review for validation studies of administrative health data from 2014 was able to identify only a very limited number of pediatric validation studies for a wide range of diseases and revealed that most studies focused exclusively on adult populations (44).

When working with larger sample sizes in administrative data research, issues related to statistical analyses may occur. For instance, although associations between outcome variables and exposures present as statistically significant (*p* < 0.05) due to a large sample size and power, clinical significance is not always evident and should be critically evaluated when reviewing the results (25). The clustered nature of the data sets in administrative health databases may also result in overestimating associations, which may lead to incorrect conclusions, and ideally should be addressed through models that take into account the hierarchical nature of data present (11).

Just like any other type of study, administrative health data research has the potential to be affected by information bias. Misclassification of an outcome can result when there is unclear or unavailable clinical documentation or a range of definitions for a certain disease, as mentioned previously (16). To address this, adding additional data sources through data linkage can be performed (16). Confounding by indication (also known as indication bias) commonly occurs when people with certain conditions are more likely to get certain tests or diagnoses attributed to them (45). For example, if someone already has AKI, they may be more likely to be diagnosed with CKD, but this does not necessarily mean they were originally at a higher risk for having CKD.

Additionally, selection bias may result when certain diagnostic codes do not selectively represent a condition under study as study samples will be restricted to a special population, especially when consent is required (11). Careful planning of studies should be done to avoid these biases. When carrying out different objectives during an administrative health data–based research study, using a multidisciplinary team of researchers that include statisticians with expertise in health data analysis and modeling of the specific database being used is essential (11, 39).

## Validation Studies for Administrative Health Care Data in AKI

Since the late 1990s, there has been increasing interest in validating the use of discharge and procedural billing codes from administrative health care data research on AKI, including severe AKI requiring kidney replacement therapy (KRT) (46). In 2011, Vlasschaert et al. published a systematic review on the validity of using administrative codes for AKI and CKD (47). The results of this review showed high specificity but lower sensitivity for identifying AKI using administrative data; of note, sensitivity is improved when administrative health care data are used to identify more severe AKI and/or AKI treated with KRT. We conducted a MEDLINE search with the following search terms: (1) *acute kidney injury, aki, acute renal failure*, or *continuous renal replacement therapy*, and (2) admin^*^ adj3 data^*^ to identify validation studies published since this systematic review, which identified an additional eight studies. A summary of the results of these studies (48–55) and a selection of those published in the systematic review (56–61) are included in Table 2. To our knowledge, this represents the most up-to-date summary of validation studies for AKI administrative health research.

**Table 2:**
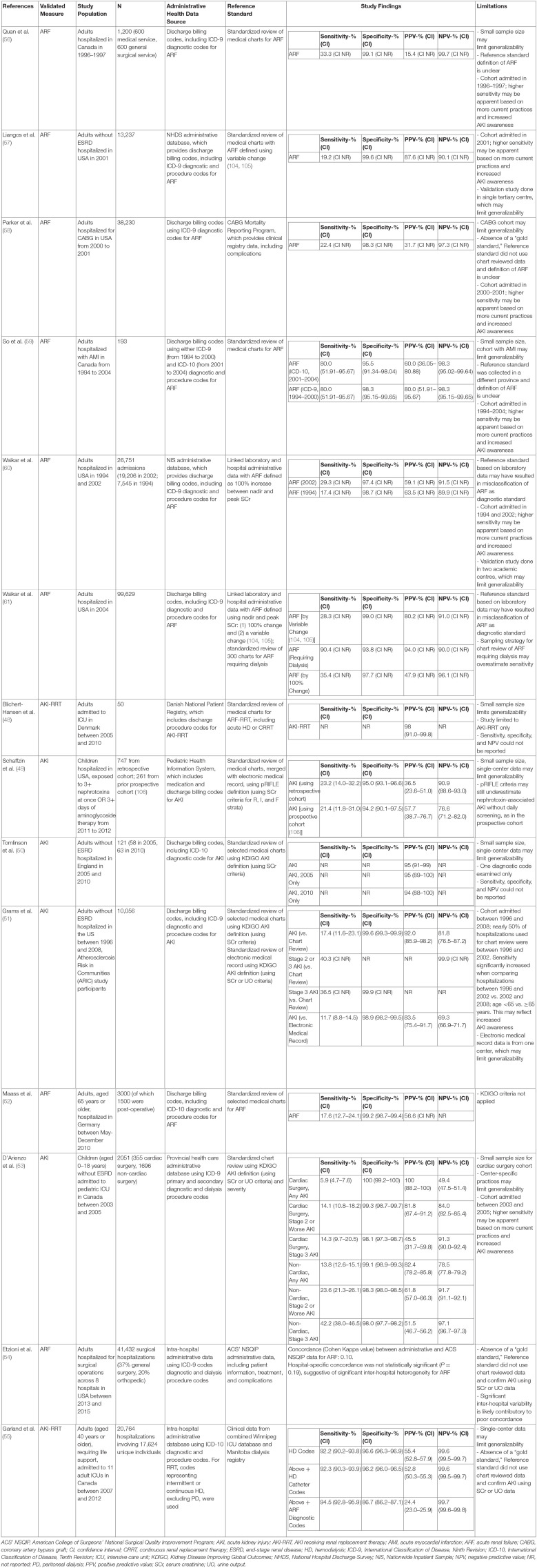
Summary of validation studies for acute kidney injury and renal replacement therapy codes in adults and pediatric administrative health research.

*ACS' NSQIP, American College of Surgeons' National Surgical Quality Improvement Program; AKI, acute kidney injury; AKI-RRT, AKI receiving renal replacement therapy; AMI, acute myocardial infarction; ARF, acute renal failure; CABG, coronary artery bypass graft; CI, confidence interval; CRRT, continuous renal replacement therapy; ESRD, end-stage renal disease; HD, hemodialysis; ICD-9, International Classification of Disease, Ninth Revision; ICD-10, International Classification of Disease, Tenth Revision; ICU, intensive care unit; KDIGO, Kidney Disease Improving Global Outcomes; NHDS, National Hospital Discharge Survey; NIS, Nationwide Inpatient Sample; NPV, negative predictive value; NR, not reported; PD, peritoneal dialysis; PPV, positive predictive value; SCr, serum creatinine; UO, urine output*.

We have summarized 14 studies that took place between 2004 and 2020. Validation studies have generally compared cases of AKI identified from administrative data, including diagnostic and/or procedure codes for AKI, to a reference standard. More recent validation studies tended to use *ICD-10* codes (50, 52, 55, 59), although many studies utilized *ICD-9* codes (51, 53, 54, 56–61). For the most part, the reference standard has consisted of either standardized chart review (48, 50–53, 56, 57, 59) or laboratory data linked to either an (1) electronic health record (49, 51) or (2) administrative database containing data from inpatient and outpatient laboratories (54, 55, 58, 60, 61). The source for the reference standard significantly impacted study size with full chart reviews tending to have much smaller study populations. Early studies used acute renal failure (ARF) (56–61) for the reference standard, but the definition of ARF was not always well-defined. Newer studies have tended toward using KDIGO criteria (62) for AKI using serum creatinine levels as the reference standard (50, 51, 53); two studies used serum creatinine or urine output criteria (51, 53). Several studies compared only patients with more severe AKI against the reference standard (48, 51, 53, 55). Most studies were done in hospitalized adults (48, 50–52, 54–61); two studies examined adults admitted to the ICU (48, 55), and two early studies examined adults post–coronary artery bypass graft (58) operation and post–acute myocardial infarction (59). Only two studies were done in children (49, 53); one study examined hospitalized pediatric patients exposed to nephrotoxins (49) and the other examined pediatric patients admitted to the ICU (53).

Most studies reported various parameters, such as sensitivity, specificity, positive predictive value (PPV), and negative predictive value (NPV) of the administrative data vs. reference data for identifying cases of AKI or ARF. Specificity of administrative data tended to be high (86.7–100%). Studies comparing administrative data to a standardized chart review, regardless of definition used, had a high specificity (≥94%) (48–53, 56, 57, 59–61). Two studies compared administrative data to another administrative database and reported reduced specificity (86.7–98.3%); however, these results are likely to be impacted by the quality of data in the administrative database used as a reference standard and are less reliable. However, sensitivity tended to be highly variable (5.9–94.5%). Studies with higher severity of AKI or AKI receiving KRT or ARF generally reported a higher sensitivity. When compared to standardized chart review as the reference standard (51, 53), administrative data identified patients with stage 3 AKI by KDIGO criteria with a sensitivity of 36.5% in hospitalized adults (51), 14.3% in pediatric cardiac surgery patients in ICU (53), and 42.2% for pediatric non-cardiac surgery patients in ICU (53). In one study of billing codes of adult hospitalizations, applying criteria for creatinine or urine output did not improve sensitivity of administrative health care data, relative to chart review, using creatinine only (sensitivity 11.7 vs. 17.4%) (51). Of note, the authors used KDIGO criteria for creatinine, but a more stringent definition for urine output, to improve sensitivity. In another study of children admitted to ICU, sensitivity was also not improved when using creatinine or urine output criteria, relative to creatinine alone (53). However, urine output criteria alone, relative to creatinine alone, had higher sensitivity for detecting any AKI (13.7 vs. 5.9%) and for detecting stage 2 or worse AKI (18.2 vs. 14.1%). NPV was more variable (49.4–99.7%), but most reported an NPV of >80%. PPV was more variable (24.4–100%), but most reported PPV of >50%. These results are consistent with those published in an earlier systematic review (47).

There are a number of limitations when validating administrative data for AKI. The most significant problems are related to the fact that the definition of AKI has changed significantly in the past 15 years, which is certainly a source of misclassification bias of AKI outcomes (16). Although oliguria has been associated with increased mortality in ICU populations and is an important component of the KDIGO definition (3, 63), urine output data are also difficult to extract from administrative databases and even chart review (51). Most validation studies have used creatinine criteria alone. Further validation studies are required to validate the use of billing codes using KDIGO criteria, different stages of severity, and inclusion of serum creatinine and/or urine output criteria for use in administrative health research.

There are additional challenges with validity when interpreting data based on physician billing codes. Physician billing practices change as our understanding of the significant morbidity and mortality associated with AKI evolves over time. For example, it is unclear whether practice changes are responsible for the increasing incidence of AKI observed from administrative health studies; however, the only study comparing administrative data at different time periods (2005 vs. 2010) did not show any significant change in PPV over time (50). Many administrative databases report only a limited number of billing codes; therefore, patients with milder AKI may be underrepresented. As well, billing codes have continued and will continue to change over time, requiring repeated validation of newer codes (16, 64). However, one study comparing administrative data derived from *ICD-9* vs. *ICD-10* billing codes showed no change in sensitivity (80.0%) and only a small decrease in specificity (98.3 vs. 95.5%), respectively (59).

Another major issue is limited generalizability, or external validity, given the significant heterogeneity of these studies (16). For example, the study by D'Arienzo et al. suggests that sensitivity may be lower in pediatric patients with milder AKI and/or post–cardiac surgery (53). The study by Schaffzin et al. suggests that sensitivity is lower in pediatric patients with nephrotoxin-related AKI (49). These results reflect the significant heterogeneity in the populations studied, including age, disease severity, and etiology of AKI. Furthermore, practice standards are highly variable for the recognition and management of AKI, and this may be different across countries or academic vs. community centers. The reference standard is usually based on standardized chart review at one to two academic centers; these results may not be generalizable to nationwide practice for patients with AKI. Finally, administrative databases may not capture certain segments of the population in countries where there is both universal public and private health care systems and/or large uninsured populations (16).

In summary, these validation studies continue to be limited by variable definitions for AKI, significant heterogeneity of data sources and population studies, and reduced generalizability. Future studies are needed to further validate subpopulations, particularly in the pediatric population; as well, standardized guidelines for defining AKI using discharge billing codes will be essential in improving the applicability of administrative data to AKI health research (19).

## Epidemiology and Outcomes of AKI

Administrative health research has served as an important methodological source for a number of observational epidemiology studies, which have fundamentally changed our understanding of AKI. This section will focus on studies in AKI conducted using administrative health data in the past 5 years.

### Incidence

AKI is common in hospitalized patients with particularly high incidence rates seen in critically ill, post–cardiac surgery, oncology, and nephrotoxin-exposed adults and children (1, 3, 62, 65, 66). However, there have been conflicting results regarding the changing temporal trends in AKI and AKI receiving KRT. One of the first studies used a national database to identify cases of AKI receiving KRT using *ICD-9* codes and found that from 2000 to 2009, the incidence had increased by an average of 10% per year (67). Another study identified more than 18,000 adult patients with AKI receiving KRT using *ICD-10* codes between 2000 and 2012 (68). The authors found that the crude incidence rate of AKI receiving KRT increased nearly 3-fold from 2000 to 2006; although the rate of growth remained stable between 2006 and 2012, the use of continuous renal replacement therapy (CRRT) increased throughout this period and especially in patients >75 years with high comorbid disease. In children, a similar trend of dialysis receiving AKI has been reported (69). Our group found an increasing trend in the incidence of AKI, as well as use of hemodialysis and CRRT among hospitalized children (1 month to 18 years) in Ontario, Canada (69). However, another large study, which compared the annual AKI incidence rate using data from an electronic health record surveillance tool and administrative data (70), found no difference in AKI incidence between 2006 and 2014 when adjusted for age and sex using either data source.

### Short-Term Outcomes

Health administrative databases have improved our understanding that AKI is independently associated with increased hospital mortality and morbidity, including length of hospital and ICU stay, and increased health care costs. One study in Japan across more than 280 hospitals showed a decreasing trend in crude in-hospital mortality from 45% in 2007 to 36% in 2016 (71). An Italian study showed in-hospital mortality rate of nearly 30% with the highest risk being in patients with AKI receiving KRT [odds ratio, 2.7; 95% confidence interval (CI), 2.7–2.8] (72). Finally, another Canadian study showed mortality rates between 30 and 40% for adult patients with AKI receiving KRT, but no association with increased mortality in centers who manage a lower volume of patients requiring KRT (73). The Canadian study in children with AKI receiving KRT also examined 30-day mortality, finding an increased rate from 14 to 25% between 1996 and 2009, although this rate subsequently decreased to 19% by 2015 (69). Another study in Japan included pediatric patients (>12 years) and reported 50% in-hospital mortality rate for patients treated with CRRT (74).

Several studies have also described rates of renal recovery, as well as the recurrence risk after AKI, both of which reflect a growing appreciation for AKI as a dynamic process (75–78). A study of critically ill children in Canada showed that children with stage 3 AKI were more than 3-fold likely to have elevated serum creatinine at discharge (>1.5 × baseline), relative to those without stage 3 AKI (79). A small study examined children who received ventricular assist devices (VADs) for heart failure and subsequently went on to undergo heart transplant (80). Those children without renal recovery (serum creatinine ≥1.5 × baseline) 7 days after VAD implantation, relative to children who had full recovery, were not at increased risk for CKD at 1 year following heart transplant; however, those with reduced estimated glomerular filtration rate 1 month after VAD implantation were at increased risk for CKD.

There is also increased interest in the relative cost and burden associated with AKI. One study in Alberta, Canada, estimated the incremental cost of AKI to be more than Canadian $200 million per year (81). Patients with AKI receiving KRT had prolonged length of stay by more than 7 days and increased cost by up to $20,000, relative to patients without AKI. Another large study of adults from Alberta, Canada, undergoing cardiac surgery also showed increased length of stay and costs associated with increased AKI severity (82).

### Risk Factors

Risk factors for AKI in hospitalized patients include extremes of age, underlying illness (i.e., sepsis, cardiovascular disease with or without bypass surgery, oncologic disease), nephrotoxin exposure, inflammatory mediators (i.e., cytokine release), and disease severity (62, 75, 83–89). A large study in the United States of 3.6 million postsurgical patients found that AKI was common postoperatively, affecting more than 10% of hospitalized patients (86). AKI in patients with CKD is also an important risk factor for subsequent progression to ESKD (88). One study highlighted that patients who progressed from stage 3 to stage 4 CKD also had a high risk for AKI (89). Other subpopulations noted to be at increased risk for AKI-related morbidity and mortality include adults with decompensated liver disease (90) and stroke (91).

A number of studies have examined the impact of nephrotoxic medications using administrative health data (92–98). One study in hospitalized children across six of the largest children's hospitals in the United States found that combined use of vancomycin with piperacillin/tazobactam conferred more than 3-fold increased risk for antibiotic-associated AKI, relative to vancomycin with another antipseudomonal antibiotic (93). Other nephrotoxins examined include non-steroidal anti-inflammatory drugs in young, healthy adults (94), brands and dosing of immunoglobulin products (95), and statin use in elderly patients (96), all of which showed modest associations with AKI. Administrative data were also used to develop an evidence-based nephrotoxin medication list that could be used as part of a screening program for AKI in children (98).

### Long-Term Outcomes

AKI is associated with increased risk for development of cardiovascular disease, CKD, and ESKD in adults (1, 75, 78, 83, 99). A study in Northern California, including more than 43,000 patients, showed that AKI in adults is independently associated with HTN as early as 6 months after initial event (99). Another large study of more than 100,000 patients in the United States showed that more than 30% of hospitalized adults with AKI went on to develop CKD at 1-year follow-up (78).

The risks of long-term kidney and cardiovascular sequelae after pediatric AKI remain uncertain, at least partially due to the fact that studies have lacked comparator cohorts, have had high losses to follow-up, and/or have had short follow-up periods. Recent prospective cohort studies have conflicting results; TRIBE-AKI, FRAIL-AKI, and ASSESS-AKI all found that cardiac surgery–associated AKI survivors have similar 4–7-year kidney outcomes vs. children without AKI (24–26), whereas Benisty et al. found higher long-term risks of CKD and HTN among survivors of ICU-associated AKI (27). Although there is a clear signal of increased CKD risk among pediatric AKI survivors in other cohort studies (6, 13, 28–30), the long-term outcomes after episodes of dialysis-receiving AKI remain uncertain.

Health administrative databases provide a unique opportunity to follow children many years after an episode of AKI, even after the age of 18 years, to study their long-term outcomes. Moreover, CKD and HTN-related cardiovascular changes start early in life; therefore, it is essential to understand the timing and magnitude of the onset of these events after an episode of AKI, so that appropriate treatment can be initiated in time to address these risk factors and avoid or delay future cardiovascular disease (100). Current AKI guidelines in neonates and children do not provide recommendations for long-term follow-up, primarily due to a lack of studies with robust data.

Several studies have examined long-term outcomes in hospitalized children with AKI. A recent study from Ontario using administrative health data with median 10-year follow-up compared children surviving AKI receiving KRT with hospital-matched controls (101). The authors found that these children had significantly increased hazard for major adverse kidney events (composite outcome of kidney failure and all-cause mortality) [adjusted hazard ratio (HR), 4.97; 95% CI, 4.04–6.10], CKD (adjusted HR, 8.70; 95% CI, 6.68–11.34), and HTN (adjusted HR, 3.35; 95% CI, 2.59–4.33). Another cohort of critically ill children with AKI in Quebec was also found to have increased risk for mortality 5–7 years following hospital discharge, relative to children without AKI (adjusted HR, 3.1; 95% CI, 1.5–6.6) (6). Health care utilization was also increased for critically ill children with AKI, relative to children without AKI, including increased hospitalizations and physician visits at 5 years following hospital discharge (7). This cohort also had increased risk for HTN (adjusted HR, 2.19; 95% CI, 1.47–3.26) (9) and CKD (adjusted HR, 2.2; 95% CI, 1.1–4.5) (8) at 5-year follow-up, defined using administrative health care data. Another study following critically ill children with congenital heart disease showed that those who developed AKI within 5 days of cardiac surgery were at significantly increased risk for CKD at 5 years, relative to those who did not develop AKI (HR, 3.8; 95% CI, 1.4–10.4) (102). We have also previously reported that, among children who underwent cardiac surgery for congenital heart disease, those who received dialysis for AKI during their index cardiac surgery admission were at a 5-fold higher risk of ESKD (crude HR, 5.0; 95% CI, 2.0–12.6) compared with those who did not receive dialysis during their cardiac surgery admission (103).

## Conclusion

Our understanding of AKI has undergone a significant transformation over the past 15 years partly due to methodological advances in which AKI has been studied by epidemiologists (1). Hand-in-hand with a veritable explosion of research demonstrating the morbidity and mortality related to AKI in adults and children, there has been significant attention paid to “big data” (18, 25, 37). Registries containing administrative health care data are readily available, efficient, and large (14, 19). Validation studies have been shown that administrative health care data are highly specific, despite having modest sensitivity, for the diagnosis of AKI. There are important limitations as well, including heterogeneity of data sources and inconsistent reporting on specific outcome measures (i.e., quality of life, patient-reported outcomes). The statements from the ADQI consensus conference recognized many strategies by which administrative health data could be applied to AKI-specific research studies and address knowledge gaps in the field (18–23).

Studies using administrative health care data have expanded our understanding of the incidence, risk factors, and outcomes of AKI in both adults and children. Importantly, we have demonstrated that these clinically relevant findings have been supported by results from traditional data sources. Continued progress in the application of large data sources to epidemiological studies will continue to further our understanding of important patient-related outcomes of adults and children with AKI.

## Author Contributions

EU drafted the manuscript, prepared the figures, and reviewed and revised the manuscript. GS also contributed to the manuscript and reviewed and revised the manuscript. RC supervised the manuscript preparation and reviewed and revised the manuscript for intellectual content. MZ reviewed and revised the manuscript for intellectual content. All authors approved the final manuscript as submitted.

## Conflict of Interest

The authors declare that the research was conducted in the absence of any commercial or financial relationships that could be construed as a potential conflict of interest.

## Publisher's Note

All claims expressed in this article are solely those of the authors and do not necessarily represent those of their affiliated organizations, or those of the publisher, the editors and the reviewers. Any product that may be evaluated in this article, or claim that may be made by its manufacturer, is not guaranteed or endorsed by the publisher.
